# Analysis of the low density lipoprotein receptor gene (LDLR) mutation spectrum in Russian familial hypercholesterolemia

**DOI:** 10.18699/VJGB-22-38

**Published:** 2022-05

**Authors:** V.B. Vasilyev, F.M. Zakharova, T.Yu. Bogoslovskaya, M.Yu. Mandelshtam

**Affiliations:** Institute of Experimental Medicine, St. Petersburg, Russia; Institute of Experimental Medicine, St. Petersburg, Russia; Institute of Experimental Medicine, St. Petersburg, Russia; Institute of Experimental Medicine, St. Petersburg, Russia

**Keywords:** familial hypercholesterolemia, low density lipoprotein receptor gene, mutations, семейная гиперхолестеринемия, рецептор липопротеинов низкой плотности, мутации

## Abstract

Familial hypercholesterolemia (FH) is a very common human hereditary disease in Russia and in the whole world with most of mutations localized in the gene coding for the low density lipoprotein receptor (LDLR). The object of this review is to systematize the knowledge about LDLR mutations in Russia. With this aim we analyzed all available literature on the subject and tabulated the data. More than 1/3 (80 out of 203, i. e. 39.4 %) of all mutations reported from Russia were not described in other populations. To date, most LDLR gene mutations have been characterized in large cities: Moscow (130 entries), Saint Petersburg (50 entries), Novosibirsk (34 mutations) and Petrozavodsk (19 mutations). Other regions are poorly studied. The majority of pathogenic mutations (142 out of 203 reported here or 70 %) were revealed in single pedigrees; 61 variants of mutations were described in two or more genealogies; only 5 mutations were found in 10 or more families. As everywhere, missense mutations prevail among all types of nucleotide substitutions in LDLR, but the highest national specificity is imparted by frameshift mutations: out of 27 variants reported, 19 (or 70 %) are specific for Russia. The most abundant in mutations are exons 4 and 9 of the gene due to their largest size and higher occurrence of mutations in them. Poland,the Czech Republic, Italy and the Netherlands share the highest number of mutations with the Russian population. Target sequencing significantly accelerates the characterization of mutation spectra in FH, but due to the absence of systematic investigations in the regions, one may suggest that most of LDLR mutations in the Russian population have not been described yet.

## Abstract (ru)

Семейная гиперхолестеринемия – распространенное во всем мире наследственное заболевание человека, при котором чаще всего дефекты обнаруживаются в гене рецептора липопротеинов низкой плотности (LDLR). Цель работы – систематизировать знания о мутациях гена LDLR в России. Проведен анализ литературы по предмету исследования, составлены сводные таблицы, показывающие встречаемость мутаций в отдельных регионах, и определены часто встречающиеся мутации. Более трети (80 из 203, т. е. 39.4 %) патогенных или вероятно патогенных мутаций представлены вариантами, специфичными для России и не встречающимися в других странах. Наибольшее количество вариантов охарактеризовано в крупных городах: Москве (130 патогенных мутаций), Санкт-Петербурге (50), Новосибирске (34) и Петрозаводске (19), тогда как регионы охарактеризованы гораздо хуже. Подавляющее число патогенных мутаций (142 из 203, или 70 %) найдено в единичных семьях, и только 61 вид мутаций встречался в двух или в нескольких родословных. Лишь 5 видов мутаций были найдены не менее чем в 10 семьях. Как и везде в мире, в России в гене LDLR преобладают миссенс-мутации, но особенным национальным своеобразием характеризуются мутации типа сдвига рамки считывания: из 27 найденных вариантов 19 (70 %) специфичны для России. Наивысшее число мутаций в гене LDLR в российской популяции обнаружено в четвертом и девятом экзонах. Это определяется тем, что четвертый и девятый экзоны являются самыми протяженными в гене и кодируют функционально важные участки белка, что обусловливает повышенную плотность патогенных мутаций в расчете на один нуклеотид длины именно в этих экзонах. Российская популяция имеет наибольшее число совпадающих мутаций с популяциями Польши, Чешской Республики, Нидерландов и Италии. Внедрение методов таргетного секвенирования существенно ускорило характеристику мутационного спектра при семейной гиперхолестеринемии, но из-за отсутствия систематических исследований в регионах большинство видов мутаций в России, вероятнее всего, еще не описано.

## Introduction

The term ‘familial hypercholesterolemia’ (FH) is generally used to refer to monogenic diseases caused by mutations in the low-density lipoprotein (LDL) receptor (LDLR) gene (OMIM 606945), in the apolipoprotein B (APOB) gene (OMIM 107730), in the PCSK9 gene (OMIM 607786), in the adapter protein gene for the LDL receptor LDLRAP1 (OMIM 605747) and some minor genes, such as STAP1, APOE, LIPA, or in the sterol transporter genes, sterolins ABCG5/ABCG8 (Defesche et al., 2017; Berberich, Hegele, 2019). At the same time, 80–85 % of FH cases are caused by mutations in the LDL receptor gene. Mutations in the apolipoprotein B gene are responsible for 5–10 % of FH cases. Mutations in the PCSK9 gene and in the LDL receptor adapter protein gene are the rarest, occurring in no more than 1 % of patients with FH.

It was previously believed that heterozygous FH occurs in 1 out of 500 people examined in the population, but the current data allow us to conclude that it is much more frequent. A study of 69,106 patients in Denmark who were diagnosed with FH based on the recommendations of the Dutch Lipid Clinic Network (DLCN) demonstrated that the prevalence of the disease is 1:219 (Benn et al., 2012). It may be even higher in Russia, i. e. 1:148 (Ershova et al., 2017). However, in this instance, cases of not only definite, but also probable FH were taken into account. Such frequency allows attributing FH to the most common monogenic human diseases

Already in 2018, the ClinVar database (Landrum et al., 2016) included 4973 variants of the LDLR gene (Iacocca et al., 2018) associated with FH, of which 2351 variants were classified as pathogenic, and 1525 as probably pathogenic, the rest considered as benign variants or variants of uncertain clinical significance. The history of FH research in Russia has recently been reviewed (Vasilyev et al., 2020; Meshkov et al., 2021a). Most of the mutations leading to FH, as expected, were found in the LDLR gene, 187 pathogenic or likely pathogenic variants of which were identified in Russia (Meshkov et al., 2021a); 67 out of 187 were not described in other populations of the world. An important article on the genetics of FH in St. Petersburg was later published based on targeted sequencing of genes involved in the origin of the disease (Miroshnikova et al., 2021). As a result, 23 variants of the LDLR gene sequence were found in the St. Petersburg population, most of which had not been described in that area (Mandelshtam et al., 1993; Tatishcheva et al., 2001; Zakharova et al., 2005, 2007; Vasilyev et al., 2020). The results of studying mutations in the regions of Russia appeared only recently (Meshkov et al., 2021b). Continuously replenished data on the subject indicate the necessity for regular revisions of the tables of the LDLR gene mutations in Russia (Meshkov et al., 2021a). In our view, such a notion supports the relevance of the present review: it already mentions 203 pathogenic or likely pathogenic variants in the gene discussed

## Methods

All available literature concerning LDLR gene mutations in the Russian population was analyzed. As a result, a summarizing table was compiled that significantly expands our knowledge about the spectrum of mutations in Russia, as compared to previously published data (Mandelshtam et al., 1993, 1998a, b; Chakir et al., 1998a, b; Krapivner et al., 2001; Mandelshtam, Maslennikov, 2001; Tatishcheva et al., 2001; Zakharova et al., 2001, 2005, 2007; Meshkov et al., 2004, 2009, 2021a, b; Voevoda et al., 2008; Komarova et al., 2013a–c; Korneva et al., 2013–2016, 2017a, b; Shakhtshneider et al., 2017, 2019a, b, 2021; Averkova et al., 2018; Semenova et al., 2020) (Supplementary Table)


Supplementary Materials are available in the online version of the paper: http://vavilov.elpub.ru/jour/manager/files/Suppl_Vasilyev_engl.pdf


The term ‘mutation’, used throughout the text, implies all rare variants of a gene (widespread polymorphisms excluded) that are potentially capable of causing a disease, including the variants with proven or highly probable pathogenicity. Synonymous substitutions are not considered in this review (their list is presented in Vasilyev et al., 2020).

## Results and discussion

**Fig. 1.**
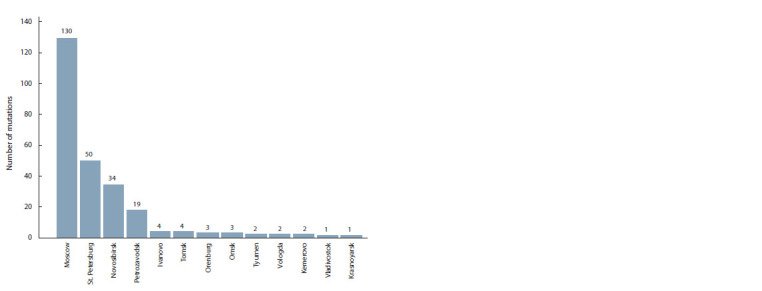
The number of pathogenic and likely pathogenic variants in the LDLR gene found in Russian cities with examined populations (excluding common polymorphisms and benign variants).

There are more than 4900 variants of the LDLR gene described in the world, as was already mentioned (Iacocca et al., 2018). This review reports 203 pathogenic or likely pathogenic mutations of this gene in Russia (see Suppl. Table). However, this diversity is not likely to represent the variability of the receptor gene in the Russian population, since GWS was introduced somewhat recently, and a systematic study of the FH genetics in quite a few regions of Russia has not been conducted. The studies were carried out mainly in large cities (Fig. 1). Currently most of the mutations found are specific for each of these cities, and a significantly smaller proportion is common with other regions. Thus, the largest number of pathogenic or probably pathogenic variants in Russia (101) was found only in Moscow (Krapivner et al., 2001; Meshkov et al., 2004, 2009, 2021a; Averkova et al., 2018; Semenova et al., 2020), 35 variants were found only in St. Petersburg (Mandelshtam et al., 1993; Zakharova et al., 2001, 2005, 2007; Tatishcheva et al., 2001), 23 – only in Novosibirsk (Voevoda et al., 2008; Shakhtshneider et al., 2017, 2019a, b), 11 – only in Petrozavodsk (Komarova et al., 2013a–c; Korneva et al., 2013, 2014, 2017a, b), 33 – in other regions, sometimes in several regions simultaneously (Meshkov et al., 2021b).

At the moment, we can state a wide variety of mutations in Russia, of which more than one third (39.4 %) are specific for the local population and have not been found anywhere else in the world so far (Table 1). The distribution of mutations by type in Russia is very similar to that in the world (see Table 1).

**Table 1.**
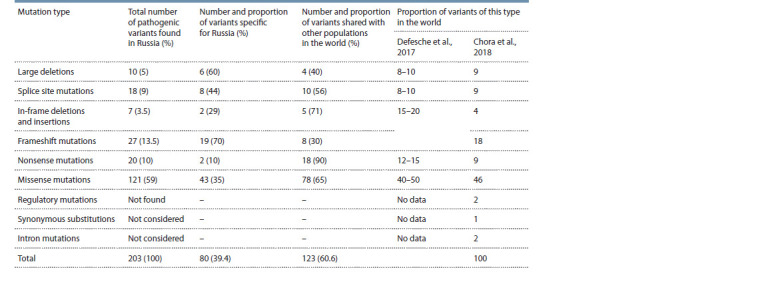
Analysis of the spectrum of pathogenic and likely pathogenic mutations of the LDLR gene in Russia and in the world

From the analysis of Table 1 it follows that the underestimated percentage of large deletions is due to the fact that targeted sequencing was introduced only recently, and a targeted large-scale search for large deletions was not carried out: the researchers focused on exon screening, which determined a slightly higher percentage of missense mutations than in the world as a whole. The systematic search for large deletions in the LDLR gene began very recently (Shakhtshneider et al., 2021). It included patients with FH in whom high-throughput targeted sequencing did not reveal significant mutations in a panel of 43 lipid metabolism genes using multiplex ligase-dependent PCR (MLPA), which revealed two deletions of the LDLR gene in a studied sample of 80 patients with FH.

**Fig. 2.**
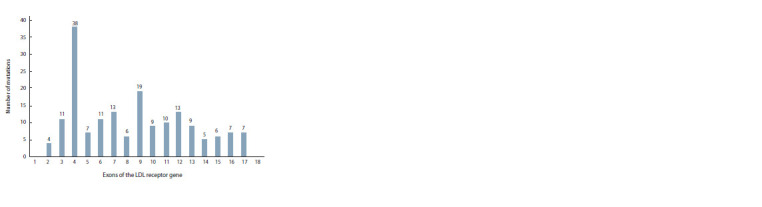
Distribution of pathogenic and probably pathogenic variants by exons in the LDL receptor gene in patients with FH in Russia

Only a few variants of the LDLR gene occur in several families, but unique mutations predominate. The majority of pathogenic mutations (142 out of 203 or 70 %) in Russia were also found in singular families, and only 61 types of mutations were found in two families or in more pedigrees. Around the world, the largest number of mutations is described in the 4th exon. Firstly, it is the largest of all exons in the LDLR gene. Secondly, it has the highest density of mutations, amounting to 0.882 variants per nucleotide (Chora et al., 2018). It is in this exon that the largest number of functionally characterized mutations was found, and almost all of those have a pathogenic effect. Our study showed that the largest number of mutations in the LDLR gene in the Russian population is localized in the largest exons, i. e. the 4th and 9th (Fig. 2). Considering all the information available, we conclude

Only five pathogenic variants of the LDLR gene in Russia can be classified as major, found in 10 or more families (Table 2). Of these, only one is specific for Russia, while the rest are widespread in the world.

**Table2.**
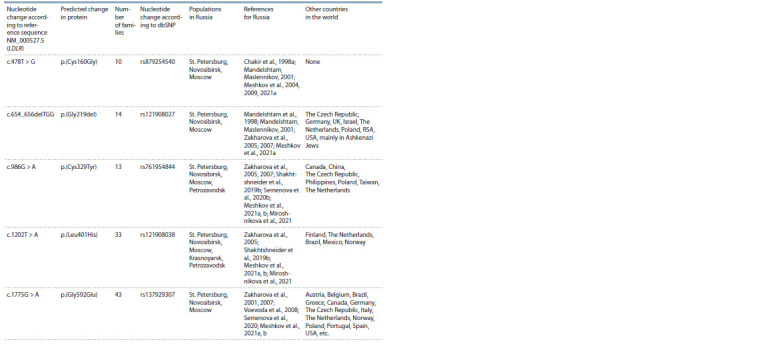
Pathogenic variants in the LDLR gene that were found in patients with FH from the Russian population in 10 or more families

The greatest similarity in the spectrum of mutations in the LDLR gene in Russia is observed with Poland, the Czech Republic, the Netherlands, Spain and Italy, which is partly determined by the fact that these populations are the best characterized (Table 3). This similarity probably results from the presence of widespread Caucasian race mutations in the world, but is not due to migration or the founder effect.

**Table3.**
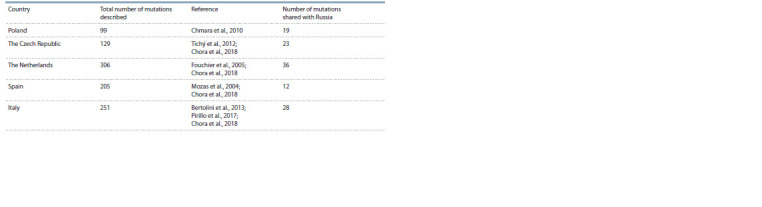
Occurrence of Russian LDLR gene mutations in other countries

## Conclusion

Thus, the success of further study of the mutation spectrum of the LDLR gene will depend on several factors, one of which is the formation of a complete nation-wide register of patients with FH, and the other is the introduction of targeted sequencing into routine practice.

## Conflict of interest

The authors declare no conflict of interest.
